# Appendagite épiploïque: cause rare d’abdomen aigu

**DOI:** 10.11604/pamj.2020.36.149.21033

**Published:** 2020-07-03

**Authors:** Saad Slaiki, Mohamed Afdil, Hicham El Bouhaddouti, El Bachir Benjelloun, Abdelmalek Ousadden, Khalid Ait Taleb, Ouadii Mouaqit

**Affiliations:** 1 Service de Chirurgie Viscérale, CHU Hassan II, Faculté de Médecine, Fès Maroc

**Keywords:** Appendagite, épiploïque, primitive, Appendagitis, epiploic, primary

## Abstract

L’appendagite épiploïque primitive est une cause rare d’abdomen aigu. Elle peut simuler le tableau clinique d’autre processus inflammatoire tels que la diverticulite ou l’appendicite. Le diagnostic repose sur le scanner. Le traitement est médical en dehors des complications.

## Abstract (en)

Epiploic appendagitis is a rare cause of acute abdomen. It can mimic other inflammatory processes such as diverticulitis or appendicitis. Diagnosis is based on CT scan. Medical management is the approach of choice in the absence of complications.

## Introduction

L'appendagite épiploïque primitive est une cause rare d’abdomen aigu. Elle survient souvent suite à des torsions et des inflammations primitives des appendices épiploïques diagnostiquées en période préopératoire. Le traitement est le plus souvent médical. A travers cette observation et une revue de la littérature nous essayons de soulever les aspects cliniques et thérapeutiques.

## Patient et observation

**Figure 1:**
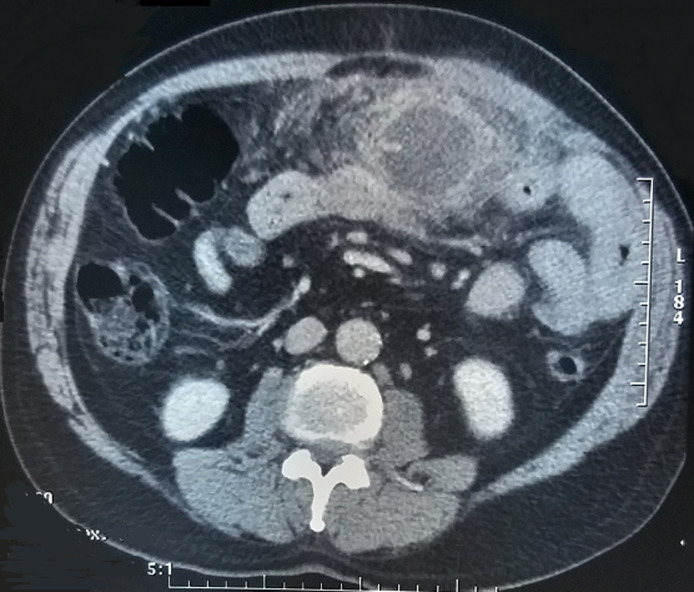
appendagite épiploïque du colon transverse compliqué d’un abcès

**Figure 2:**
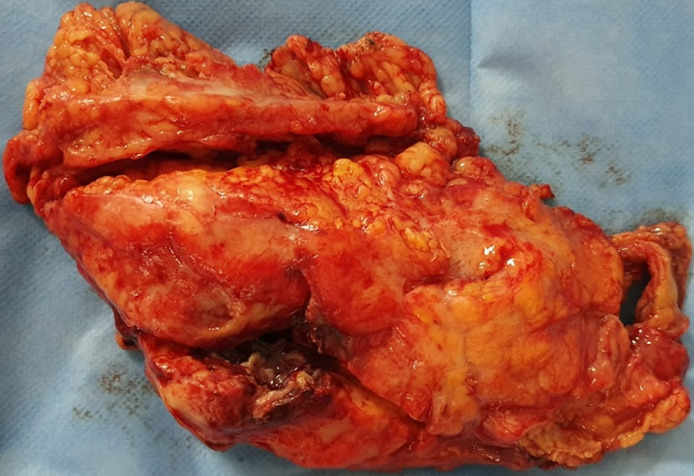
appendagite colmatée par l’epiploon

Un homme de 45 ans, sans antécédent pathologique notable. Admis aux urgences pour prise en charge d'epigastralgies, fébrile. A l’admission patient IMC à 29 conscient avec un pouls à 90 battements/min, examen abdominal a trouvé une défense épigastrique avec masse palpable le reste de l’examen somatique était sans particularité. Biologiquement des globules blancs à 20000 par millimètre cube et Une CRP à 200 mg/l. Une tomodensitométrie (TDM) était réalisée en urgence objectivant une appendagite épiploïque compliqué d´abcès (Figure 1). Patient acheminé au bloc avec découverte en peropératoire d’une frange du colon transverse abcédé et colmaté par l’epiploon le geste ayant consisté en une résection de la masse (Figure 2). Les suites étaient simples, patient sorti à j+3.

## Discussion

L'appendagite épiploïque primitive est une inflammation bénigne et spontanément résolutive des appendices épiploïques [1]. C’est une étiologie rare de douleur abdominale chez l’adulte [2]. L’incidence de cette affection n'est pas réellement connue et la prévalence est sous-évaluée, elle survient à un âge entre 20 et 50 ans avec une légère prédominance masculine [3]. Elle affecte essentiellement les sujets en surpoids, car ils ont un nombre et un volume d’appendices épiploïques plus augmenté [4]. Ces caractéristiques sont présentes chez notre patient. Les appendagites épiploïques peuvent siéger le long du colon, par ordre de fréquence, la charnière recto sigmoïdienne (57%), la région iléocæcale (26%), le côlon ascendant (9%), le côlon transverse (6%) et le côlon descendant (2%) [3]. Dans notre cas la localisation était au niveau du transverse. Cliniquement la douleur abdominale est toujours constante et localisée en fonction du siège de l’appendice pathologique, dans 10 à 30% des cas, une masse abdominale sous-pariétale est palpable [5]. Sa palpation entraine une douleur extrême. Dans notre cas c´était une masse palpable épigastrique plus latéralisée à gauche dont la palpation entraine une défense abdominale. Cependant devant cette symptomatologie le diagnostic d’appendagite est rarement posé d´où l’intérêt de l’imagerie. Le diagnostic de certitude est le plus souvent fait par la TDM sans ou avec injection de produit de contraste, mais l´échographie et l´imagerie par résonance magnétique (IRM) peuvent également être employées [6]. Sur la TDM, l’appendagite épiploïque apparaît sous forme d’une lésion ovalaire ronde et mal définie adjacente au colon ayant une densité légèrement supérieure à la graisse normale [4, 6, 7]. Le signe pathognomonique est le «ring sign» se traduit par une hyperdensité périphérique correspondant à l’inflammation de la séreuse. Toutefois l'absence de ce signe n’exclut pas le diagnostic d'appendagite épiploïque aiguë [8]. Dans la majorité des cas, l’appendagite épiploïque est de traitement médical, reposant sur le contrôle de la douleur. Les anti-inflammatoires oraux sont habituellement prescrits pendant 4-7 jours, et les antibiotiques ne sont pas souvent indiqués. La chirurgie est réservée aux patients dont les symptômes ne s'améliorent pas avec la gestion conservatrice, ainsi que ceux qui développent des complications. Dans ce cas, l’appendice enflammé sera ligaturé et réséqué [9].

## Conclusion

L’appendagite épiploïque est une cause rare d’abdomen aigu. La clinique est caractérisée par une douleur constante et localisée en fonction du siège de l’appendice pathologique. Le diagnostic de certitude repose sur la TDM. Le traitement est le plus souvent médical sauf en cas d’échec ou de complication.
